# Present and Future of the Poor-Law Infirmary

**Published:** 1913-05-17

**Authors:** 


### PRESENT AND FUTURE OF THE POOR-LAW INFIRMARY.

### Should Extra Fees be abolished?

BY A LATE INFIRMARY MEDICAL OFFICER.

The question of extra fees is an acutely controversial one. Some cry aloud for their abolition, others as vehemently plead for the maintenance of the present system of extra fees as an additional perquisite of the onerous position of medical superintendent. Extra fees are, however, a distinct bar to the administrative reform of the Poor Law. The vested interests which have grown up around this, as every other perquisite, are naturally antagonistic to their abolition. They do not even ask for compensation—they simply insist on the retention of the system. The system however, if it has not actually created any, is one that is open to abuses; and the impartial would probably vote for full-time appointments exclusive of all fees.

In Poor-Law administration there are five classes of extra fees existing at present: (1) Lunacy fees; (2) Midwifery fees; (3) Notification fees; (4) Compensation; (5) Class fees; and (6) Inquest fees. Medical superintendents are paid a definite salary plus these extra fees. Thus, for every "pauper lunatic" admitted to his institution the medical superintendent certifying the case receives an extra fee of a guinea or two. His total salary depends, therefore, on the number of cases he can bring himself to certify.

For every maternity case occurring in an institution, the superintendent or the part-time medical officer in charge of the workhouse is entitled to a fee. It is not even stipulated that he must be present at the birth. The regulations differ in different Unions, but the usual amount is half a guinea per birth.

In addition there are the usual fees for the notification of tuberculosis or infectious disease.

All the above fees are regarded by the medical superintendents as perquisites of their office. The junior officers are never considered entitled to even a fraction of them, whatever may have been their particular work with regard to the case. In some cases the case is never seen before death, but the fees are pocketed without compunction.

The salary of the medical superintendent should be a fixed one exclusive of all fees; it should be adequate, and commensurate with the work and responsibilities. The ratepayers would save on these exclusive appointments; the work would be as well done, and be free from any taint of suspicion.

### Class and Compensation Fees.

It is more difficult to decide whether these fees should be excluded or not. They refer in the first place to the fees payable by students and others for attendance on lectures given by the superintendents. As they are for purely personal work, and as the institution gains from their heads being so excellent as to attract students, this extra must be placed on a different footing, and be regarded as being due solely to personal endeavour. Fees payable by insurance and other firms for evidence given, and reports forwarded, in compensation cases may also be regarded as personal work, but as the work is attained by virtue of his official position the medical superintendent should be asked to forward a list of all such cases every quarter to the local Guardians.

The abuses to which the present system might lead are too obvious to deserve mention: that they have seldom occurred is due to the excellent work of the administrators. But why have the present system when a better scheme suggests itself? With a fixed exclusive salary, the revenue from all extra fees would be payable to the local Guardians.

